# Adolescents’ Wellbeing at School: What Helps and What Hinders From Feeling Safe and Satisfied?

**DOI:** 10.3389/ijph.2024.1607244

**Published:** 2024-12-03

**Authors:** Simona Horanicova, Daniela Husarova, Andrea Madarasova Geckova, Miriama Lackova Rebicova, Lenka Sokolova, Andrea F. deWinter, Sijmen Reijneveld

**Affiliations:** ^1^ Department of Health Psychology and Research Methodology, Faculty of Medicine, University of Pavol Jozef Šafárik, Kosice, Slovakia; ^2^ Olomouc University Society and Health Institute (OUSHI), Palacky University, Olomouc, Czechia; ^3^ Institute of Applied Psychology, Faculty of Social and Economic Sciences, Comenius University, Bratislava, Slovakia; ^4^ Department of Community and Occupational Health, University Medical Center Groningen, Groningen, Netherlands

**Keywords:** satisfaction, adolescence, wellbeing, school, qualitative study

## Abstract

**Objectives:**

The aim of this qualitative study was to identify the main factors that help and hinder adolescents’ wellbeing at school using their perspectives and experiences.

**Methods:**

We used data from 45 adolescents in the first year of high school in Slovakia (mean age = 14.98; 22.2% boys). We obtained the data using 11 semi-structured group interviews conducted in 2020/2021. Participants were selected from three types of high school with regard to graduation system. Data was analysed using consensual qualitative research and thematic analysis.

**Results:**

We identified three main themes of factors contributing to their wellbeing at school: 1. School (atmosphere and organisation of life at school, physical environment, threats and the ability of school to deal with issues); 2. Relationships (with peers and teachers, and teachers’ behaviour towards them); 3. Myself (own perceived obstacles and resilience resources).

**Conclusion:**

The organisation of life at school, surroundings, threats and dealing with issues importantly affect adolescents’ wellbeing. Therefore, adjustment of physical environment and interpersonal competences of teachers, supporting of resilience resources of adolescents should be targets for interventions and prevention programmes at schools.

## Introduction

Wellbeing of adolescents at school is an extensive, multidimensional concept which concerns experiences of positive emotions and quality of time spent within the school environment. According to the OECD (Organisation for Economic Cooperation and Development) [1] wellbeing at school includes psychological, cognitive, social and physical components necessary for a happy and satisfactory life of students. Moreover, Seligman et al. [2] describe wellbeing of adolescents as a combination of experience of happiness, emotional safety, satisfaction with work and a sense of belonging within the school environment.

There is an interconnection between students’ wellbeing and academic performance [3–5], including the achievement of educational goals and representing the level of intellectual skills and success of adolescents at school [1, 6]. Challenges may arise when focus on wellbeing at schools collides with the academic goals. Most of the attention is still focused on the academic achievement goals [7] although there are increasing efforts to pay more attention to promotion of wellbeing at schools and incorporating it into school guidelines along with pursuing academic and educational goals [8]. Taking into consideration the effect that the pursuing of said goals has on adolescents’ wellbeing and creating a balance with more fulfilling lifelong impact of positive experiences at school may, nevertheless, be equally as important [9–11].

The importance of wellbeing at school lies in providing adolescents with pleasant, safe and encouraging environment at school which is crucial in developing potential with regards to social, cognitive, moral and ethical aspects of their lives [12]. Schools represent an environment where adolescents spend a significant amount of their time gaining new knowledge and academic skills and where they experience significant social encounters as well as a place for building their resilience to inequality and developing future aspirations [13]. Previous research suggests some important school factors that may influence adolescents’ positive experiences at school. The importance of creating a caring and nurturing school environment is particularly important when it comes to adolescents’ wellbeing and positive experience at school [14, 15]. One of the most important sources of experiencing wellbeing at school is based on an interpersonal level. Relationships with teachers were proven to provide a great deal of support and concern for the students [16, 17]. Moreover, positive relationships with peers and classmates are similarly important [18] because of the feelings of acceptance. Furthermore, besides creating a caring environment, the role of schools consists in making sure that the environment for learning is safe which includes physical safety, rules, norms and discipline measures that ensure the lack of violent behaviour, bullying, threats etc. [19–22]. Preventing the occurrence of such negative experiences at school may be beneficial in averting further consequences including health compromising behaviours such as alcohol and drug abuse, psychosomatic complaints and poor mental health [23, 24].

Previous research has shown that positive experiences at school contribute to adolescents’ wellbeing and their further development, potential and health [12, 23]. Research on determinants of wellbeing at school has been previously focused on using rather quantitative methodologies without going into much depth and detail. Moreover, Slovakia is a quintessential example of a Central European country and the cultural norms, societal standards and educational system originating from the rather hierarchal constitution of Habsburg. Because of this history, different factors may contribute to the adolescents’ wellbeing at school compared to western countries that are leading examples in this area of research. Slovakia shares this with the Czech Republic, Hungary, Poland and the countries that constituted Yugoslavia formerly, making findings on Slovakia probably applicable to these other Central European countries as well. Moreover, according to the OECD [6] which also explores a wellbeing aspect of 15 years old adolescents, Slovakia has a rather low position, below the OECD average.

Studies have so far focused on the contributors to adolescents’ wellbeing at school including individual characteristics such as sociodemographic indicators e.g., socioeconomic status, gender and ethnicity, race and socialisation [25–27]; subjective indicators including personality traits, personal styles and academic skills [28, 29]. There is however a gap in an existing research lacking the evidence of adolescents’ own experiences with regards to their wellbeing at school. Therefore, the aim of this qualitative study is to identify the main factors that help and hinder adolescents’ wellbeing and safety at school using the perspective and experiences of the adolescents themselves.

## Methods

### Design of the Study

We conducted a qualitative study embedded in the international HBSC (Health Behaviour in School-aged children) study on health-related behaviour, wellbeing and social context of adolescents. First, we established a protocol and outline of the topic guide for the semi structured interviews. Second, we collected our data using semi structured interviews in a period between November 2020 until June 2021. Third, we analysed our data using the inductive method and main components and steps of the consensual qualitative research (CQR) methods and thematic analysis. CQR methodology involves the diversity of experience and opinions of each member of the research team and their ability to reach a conclusion with regards to their assumptions [30]. Data driven thematic analysis is used for the identification and analyses of topics and themes across the analysed qualitative data [31].

The study was conducted in accordance with the guidelines of the Declaration of Helsinki [32] as well as with the consolidated criteria for reporting qualitative research (COREQ) [33]. It was reviewed and approved by the Ethics Committee of the Pavol Jozef Safarik University in Kosice (19N/2020).

### Study Setting, Sampling and Participants

The sample regarded adolescents selected from the three types of secondary (high) schools in Košice, Slovakia: grammar schools, secondary schools with GCSE graduation and secondary schools with apprenticeship certificate graduation. The school system in Slovakia regarding secondary education is categorized into three main types: full secondary general education - grammar schools preparing students typically for university (with graduation certificate, which is an equivalent of A levels graduation); secondary vocational education - professional schools (with GCSE graduation and apprenticeship certificate depending on study field) focusing on practical skills and preparing students for specific career; and lower secondary vocational schools (with apprenticeship certificate graduation) providing specialized training targeting students who are not pursue for higher education. The choice of a particular type is often connected to the socioeconomic position of families. Adolescents from families with a higher socioeconomic status tend to choose secondary grammar schools while adolescents from low socioeconomic backgrounds tend to choose secondary vocational schools. In the selection of our study sample, we aimed to reach a maximum variation of respondents as well as maximum saturation of the themes and topics that we obtained [34]. We selected the schools in several steps. First, we obtained a list of all eligible high schools in Košice, Slovakia using the official Košice Self-governing Region information. Then we selected two schools from each type of the school with regard to the type of graduation (A levels; GCSE; apprenticeship certificate). Then we randomly selected two from each type of the school on the list with regard to type of graduation (A levels; GCSE; apprenticeship certificate).

Next, per school we reached and contacted the participants in several steps. First, we contacted the school administrators to inform them about the study and ask them to participate. After obtaining their consent, we contacted the parents of the students with help of the teachers and obtained parental informed consent. Thereafter, we approached the adolescents, and obtained their informed consent. All participants were informed about the voluntary participation in the study and anonymity of the provided data, and were allowed to withdraw from the study at any time. All selected adolescents were in the first year of Slovak high school, age 13–16 years. This study involved 45 participants in total (10 boys and 35 girls; mean age = 14.98).

### Procedure and Measures

We obtained the data using semi-structured online group interviews Participants got the invitation for the interview by email. Next at the start of the interview, participants were asked to fill in a questionnaire that contained the questions of age, gender, size of the place of residence, liking school, attitude towards education. Next, the interviews were conducted using a topic guide that included following questions:

1. Some children like school and other children don’t - why do you think that is? Why do some children like being at school and others don’t?
2. Many children feel that their teachers care about them. How do you know that your teacher truly cares about you?
3. Some children are quite successful at school and others not as much. How do we help those who struggle at school?
4. What should school, its teachers, students or even people from outside the school do in order to create a safe and pleasant environment?
5. What should be done (by teachers, students, outsiders) so that the time children spend in school is used effectively?

We conducted 11 group interviews that were conducted online during the second wave of COVID-19 pandemic in Slovakia. Because of COVID-19 measures, face-to-face contact with the participants was not feasible at that time. The interviews lasted between 45 and 60 min, the length depending on the number of participants for the interview, and they were recorded on video.

The online interviews were led by a trained professional in psychology who has a lot of experience with working with young adolescents in an online counselling platform. The other members of the research team performing the interviews (AMG, DH, MLR, SH, and LS) were professionals with experience in psychology and social work who participated in the interviews as silent observers. All of the members of the research team were academic research collaborants. Their personal experience and knowledge helped making a sense of the data collected and analysed. Each member of the research team was aware of their own views, values, assumptions and experiences and did their best to remain neutral and objective while handling the data. There was a social distance between the interviewer and participants induced by the COVID-19 measures and an online setting of the interviews. The anonymity and willingness to share information by the participants was maintained. To ensure the trustworthiness, the process of data collection was ensured to follow the criteria for reporting qualitative research (COREQ) [33].

### Data Handling and Analyses

In the data handling, we processed the data obtained to extract codes that we could next analyse We did so by first transcribing the interviews verbatim in the Slovak language, then checking the transcripts to ensure their accuracy and uploading them into MAXQDA standard platform for data analyses. The video records were transcribed leaving out any personal information of the participants. The further data handling and the analyses were performed by five members of the research team (SH, DH, AMG, MLR, and LS) who were all trained in CQR. First, they got familiar with the video recordings, transcripts and themes. After carefully reading each transcript, each member of the team coded the segments of the transcript individually. Next, all five members met for cross-checking and evaluation of the generated codes and coded segments of each transcript. In case of different opinions, discussion continued until the consensus was reached.

Next, for the analyses we assessed the main factors that help and hinder adolescents to perform at school, using a thematic analysis in accordance with Braun and Clarke [31] of the codes produced in the data handling. We did so by clustering codes into subthemes and themes once again done by all members of the team individually. Afterwards, the team members met for cross-checking of created subthemes and themes and discussion was held until the consensus on the final thematic map was reached.

## Results

### Description of the Characteristics of the Sample

As can be seen in Table 1, the sample consists 45 respondents aged from 13 to 16 years (10 boys and 35 girls). Almost half of the participants attended grammar school; the other half of the participants attended secondary schools graduating with GCSE or apprenticeship certificate. Most of the participants reported feeling satisfied towards school and education (like school, care about education). Almost a third of the participants reported feeling inconsistent with school and education (don’t like school, care about education).

**TABLE 1:** Descriptive characteristics of the sample (N = 45) (Slovakia, 2020–2021).

Gender	
Boys	10
Girls	35
Type of school	
Grammar school	22 (12 girls, 9 boys)
Secondary school (GCSE)	20 (girls)
Secondary school (apprenticeship certificate)	3 (2 girls, 1 boy)
School satisfaction^a^	
Satisfied	32
Inconsistent	13
Age distribution	
13 years old	1
14 years old	10
15 years old	23
16 years old	11

^a^ Satisfied-like school, care about education.

Inconsistent-don’t like school, care about education.

### Promoting and Hindering Factors to Adolescents’ WellBeing at School

**FIGURE 1:**
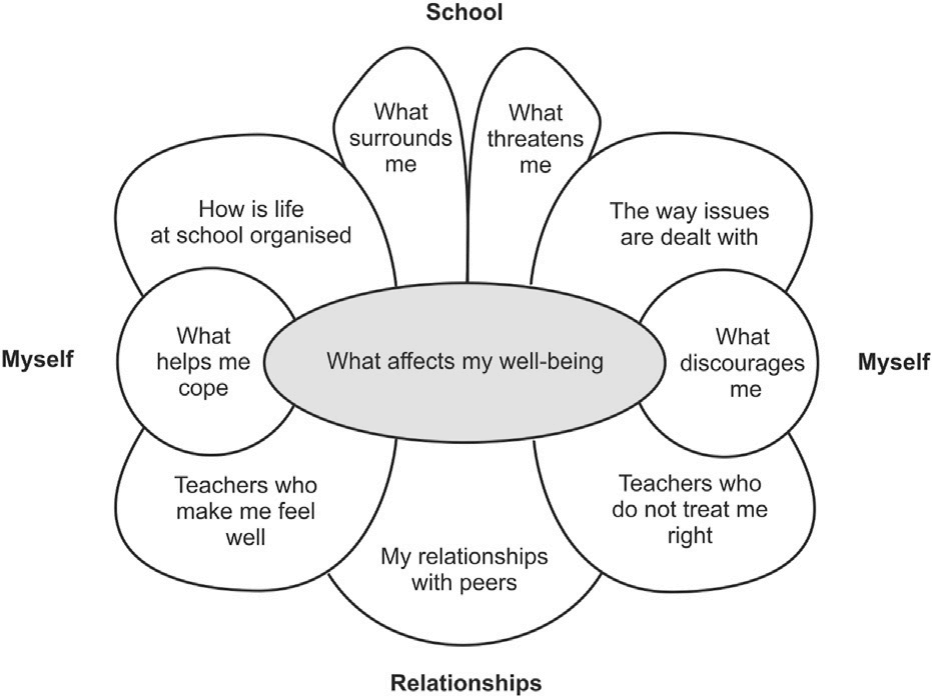
Main factors contributing to adolescents’ wellbeing at school (Slovakia, 2020–2021).

The model created using thematic analysis is shown in Figure 1 below. We identified three main themes regarding factors that contribute to adolescents’ wellbeing safety and satisfaction at school: 1. *School*, 2. “*Myself*” and 3. “*Relationships*”. Each of these themes consisted of a number of subthemes. Regarding *school,* subthemes were *how is life at school organised; what surrounds me; what threatens me; the way issues are dealt with.* Regarding *myself,* subthemes were *what discourages me* and *what helps me cope* Finally, regarding *relationships*, subthemes were *teachers who make me feel well; my relationship with peers* and *teachers who do not treat me right.* The themes and subthemes are depicted in Figure 1 and will be described in more detail below.

### School

Adolescents reported that the overall atmosphere and organisation at school are important contributors to their feeling of safety and wellbeing at school. Based on the adolescents’ statements, the need for safety and dealing with issues is considered an important factor of their wellbeing, but too much control may simultaneously make them feel trapped.

Further, adolescents reported that the overall surroundings and looks of the school along with insufficient and/or old equipment of the classrooms contribute to their dissatisfaction at school. Based on the adolescents’ statements, investments into new equipment, restoration of the premises and providing them with designated areas where they can relax may help them feel homelier and more pleasant and improve their satisfaction. Furthermore, adolescents reported that an appropriate scheduling of their classes may help them use their time spent at school effectively.

According to the statements of adolescents, experiences with perceivable threats including truancy, discrimination, racism, bullying, blackmailing, or hate speech contribute to their feeling of being threatened. On top of that, adolescents indicated that the inability of school as an institution and authorities to deal with these issues appropriately may significantly impact their safety and wellbeing. According to the statements of adolescents, competent management of the issues along with reinforcement of safety measures and teachers on duty may, on the one hand, improve their feelings of safety at school. On the other hand, adolescents indicated that too much control resulting in restricting free movement on school premises makes them feel like they are in prison.

### Myself

Adolescents stated several factors that discourage them and sources that help cope and affect their wellbeing and satisfaction at school. Adolescents, according to their statements, appreciate the routine and opportunities that school provides for them, however, the effort and demands that are put on them may often lead to feelings of discouragement.

According to adolescents’ statements, the discouraging factors include the inability of the school to fulfil their expectations along with experiencing troubles at home, waking up early in the morning, feeling strain demands on grading and difficulties with adaptation due to transfer between different school levels which affect their dissatisfaction at school.

In contrast, based on the statements of adolescents factors that help them cope include routine of school regime and providing them with activities that help build relationships with their peers, strengthen their empowerment and resilience resources and feeling more embedded within a community and significantly improve their wellbeing and school.

### Relationships

Based on the adolescents’ statements, the adolescents distinguish between the teachers who make them feel well and those who do not treat them right. Adolescents reported that the role of their relationships with teachers and peers is an important contributor to their wellbeing and satisfaction at school.

Adolescents described situations in which they experienced treatment from their teachers that was unfair and inappropriate including shouting, arguing, humiliating and abuse of power that led to feelings of fear and considerably affected their wellbeing and satisfaction at school. In contrast, based on adolescents’ statements, teachers who are attentive, bring a good mood and lighten the atmosphere significantly improve adolescents’ wellbeing.

Furthermore, adolescents reported that the teachers who maintain a non-formal approach based on mutual trust and respect and develop adolescents’ potential are a great contributor to adolescents’ satisfaction and resilience. In contrast, adolescents indicated that strong relationships with their classmates who help them fit in and feel embedded are important to their wellbeing at school. Moreover, mutually shared experiences and materials with peers at school influence their wellbeing and satisfaction at school.

Table 2 provides examples of the narratives across the themes.

**TABLE 2:** Quotes illustrating factors related to adolescents’ wellbeing and satisfaction at school (Slovakia, 2020–2021).

Themes/*Subthemes*	Quote(s)
School	
*How is life at school organised*	*“*… *For us students, some of the classes are unnecessarily late. For example, P.E. or religious education is ninth in a row, which is a common thing in our school, but we have to get back to school for that … and then you think that it’s useless to go to school because even the morning classes with more important subjects don’t give you much because you’re depressed because you have to stay late at school”…*
	*”… We should feel the authority of teachers but not excessively because that’s not good, we can’t really focus or study then, nothing. So this should be moderated. But in most cases it’s not possible because you can’t just tell the teacher to stop or not do something because that would mean we’re ill-mannered”*
*What threatens me*	*“Bullying at school. I think it’s a big issue … why the students don’t feel well. Not just the person who gets bullied but others as well for sure … because it might happen to them as well.”* *“For sure, don’t mock anyone when they struggle … Some of the classmates start to mock others when they see that they’re struggling … they start to mock them and humiliate them … there’s no need to mock anyone because they will take it close to the heart and feel even worse because of that…”*
*The way issues are dealt with*	*“So that the issues in class are dealt with. Because my former class teacher did a sloppy job, she didn’t really care and we had massive issues with bullying then, seriously, the police got involved twice, criminal complaints got filed. And she didn’t really care at the beginning, only after that and that was too much … It’s way better to deal with these things from the start because after that, if it’s excessive, nothing will get dealt with.”* *“… In the fifth grade we felt like we were in prison … We were forbidden to leave the classroom during breaks and if we walked out, we got attacked with questions like where are we going, why are we leaving and who are we going with and it came across as quite unhealthy and made us feel aversion towards school…”*
*What surrounds me*	*“… It would be nice to have some kind of room that makes us feel more at home, to spend time there during free classes. With couch or something. Somewhere where we can study … because we don’t have many classrooms and there are too many students and we have to be either outside or in corridors during free classes.”* *“… Bad environment. Badly equipped school. For example, radiators don’t work, walls are cracked, school desks and chairs are creaking.”*
Myself	
*What helps me cope*	*“Maybe if there’s an issue when others make fun of someone, it might make them feel less safe at school* … *Some discussions and talks at school might help; we used to have these at our school. They helped us know what to do in these situations and how to behave*…*”* *“*… *Maybe some kind of support or a ‘protector’ of some sort who would take them under their wings and take care of them. Or some might find it helpful to talk to someone else who went through similar things, so that they can talk it through and give each other advice.”*
*What discourages me*	*“For me it was the leap between fourth and fifth grade. The approach was different until the fourth grade, just this and that … not much was happening and then, boom, fifth grade, the cluster of several new teachers, not just one class teacher but new teachers, more schoolwork, more preparation for school … that was the leap when I thought, ‘this is not ideal’…”* *“Excessive pressure on students … For example, there are too many big tests planned for 1 day and some teachers don’t do anything about.”* *“ Maybe waking up very early in the morning … and not being able to sleep properly.”*
Relationships	
*Teachers who make me feel well*	*“When they spend more time with us. When they talk to us during breaks about old or new schoolwork topics or when they just ask about things in our life, about what we do, what have we been up to … When they are kind, sort of like parents- a mom, when a teacher has a role of a mother, when she asks how are we doing, whether there’s anything troubling us. When she’s just worried whether we’re all right … ”* *“It’s really nice when teachers don’t just start teach the second they enter the class and put their things down but when they are in a good mood … ask us how our day has been so far … ask us whether we’re healthy, whether everyone is all right … and it’s obvious that they care about us.”*
*My relationships with peers*	*“Maybe older schoolmates may let us know something like ‘Don’t worry about that, it’s just the way it works at this school. It will either get better or you’ll get used to it…’ that would make me feel safer at school. If something happens once, it may not happen again.”* *“I think that a person feels safe among strong group of people. It does not matter what the teacher is like, if the class team is strong, they can stand against her. And when people within the class team understand each other and know how to communicate, everything goes smoothly…”*
*Teachers who do not treat me right*	*“… So that they don’t humiliate someone because they don’t know something, in front of the whole class … Saying ‘It’s unbelievable that you don’t know this. You shouldn’t even be here!’…”* *“… Our teacher used to remind us that we won’t be able to pass the high school entrance exams, tests, nothing. It makes you want to give up rather than listen to this.”* *“… During middle school a teachers was biased against a boy because of how he looked, how he appeared … he wore a fringe and she just cut half of his fringe off because she said she ‘couldn’t see his eyes’…”*

## Discussion

The aim of this qualitative study was to identify the main factors that determine adolescents’ wellbeing at school using the perceptions and experiences of adolescents themselves. Using adolescents’ statements, we identified three main groups of factors including 1. *School*, 2. *Myself* and 3. *Relationships,* that contribute to their wellbeing at school. We found that the way life at *school* is organised along with surroundings, perceivable threats and dealing with issues contribute to adolescents’ wellbeing at school. *Myself* regards adolescents’ resources that help them cope and the experienced discouragements affect their wellbeing. And finally, adolescents reported that their *relationships* with peers and teachers considerably affect their wellbeing at school.

Our results show that adolescents prefer on the one hand more protection and safety at school but on the other hand, too much control makes them feel like they are in prison. Adolescents reported that experiencing threats such as bullying, discrimination or prejudice and school’s (in)ability to deal with these issues significantly affect their feelings of safety and wellbeing at school. These qualitative findings confirm previous, research showing the importance of safety at school of adolescents [35, 36]. It adds that in adolescents’ experience, safety at school is one of the most important factors of wellbeing at school which aligns with general approaches to wellbeing at school [19, 36].

Moreover, adolescents reported that the overall organisation of teaching, rules and management at school may affect their wellbeing as well. Our qualitative findings are in line with previous quantitative findings adding detailed information about the importance of the organisational justice at school and rules and their impact on students’ wellbeing [37]. Efficient organisation at school and scheduling of the classes along with appropriate applying of the rules may substantially contribute to adolescents’ wellbeing at school.

Additionally, adolescents reported that physical environment, the overall looks and equipment of school and classrooms contribute to their wellbeing and comfort at school. These qualitative results support previous quantitative research on physical features of school buildings such as design, age and students’ feelings at school [38] providing thorough information about specific features of the physical environment at school and their influence on adolescents’ wellbeing at school. Equipment that is up to date along with renovated and clean premises at school may help adolescents feel pleasant and comfortable at school. Surroundings that offer comfort, pleasant environment and effective dealing with arising issues and perceivable threats in particular, may considerably affect adolescents’ wellbeing and overall experiences at school.

Our results show that school-based daily routine, available social support and embedding within a community strengthens adolescents’ resilience resources, while difficult adaptation and too much pressure and demands at school lead to discouragements. Adolescents reported that building resilience and feeling of belonging within a community are very important with regard to their wellbeing at school. Our qualitative results support previous quantitative findings on protective factors of social context and adolescents’ resilience and wellbeing at school using adolescents’ own experiences [39, 40]. Our results provide more detailed information about adolescents’ wellbeing at school being influenced by the sense of connectedness and belonging, acceptance by their peers and good coping mechanisms [40]. In contrast, adolescents reported that experiencing personal discouragements such as transition into more formal education, harder adaptation or personal family issues may jeopardize their wellbeing and overall experiences at school. These findings align with previous findings from quantitative research showing that harder adaptation due to transition to higher, more formal education (e.g., from elementary to middle school) and personal or family issues regard risk factors related to adolescents’ resilience and wellbeing at school [40]. Our findings complement previous findings by showing that in the experience of adolescents factors on community and personal level may even more important than academic and institutional factors associated with adolescents’ school wellbeing.

We found that adolescents consider their relationships with peers and teachers an important contributor to their wellbeing at school and that they distinguish between the teachers who treat them with respect and kindness and those who treat them inappropriately. Our qualitative findings based on adolescents’ own perspectives support previous quantitative findings on the interpersonal behaviour of the teachers that show a respected but tolerant and cooperative teachers’ approach to be an important contributor to better wellbeing of the students [41]. Moreover, adolescents reported that their relationships with peers at school who help them belong and fit within the class team and whom they share their experience with are a great source of positive experiences at school. Their experiences thus confirm previous quantitative research pointing out the importance of positive interpersonal relationships among peers and classmates as one of the key protective factors and sources of resilience of adolescents at school [40, 42]. Interpersonal relationships within the school context have proven to be one of the most important factors that may affect the quality of support, engagement improving their overall wellbeing at school and school outcomes.

### Strengths and Limitations

One of the strengths of this qualitative study is the detailed exploration of the factors affecting adolescents’ wellbeing at school using their own perceptions and experiences. Moreover, using consensual qualitative research helped us avoid the individual subjective perspectives of the researchers, since every member of the research team had to agree on the codes used in coding of the data. A limitation of this study is that we may not have reached heterogeneity in the sample representative of the apprenticeship graduation students. However, during data collection no new themes appeared suggesting that saturation was reached.

### Implications

This qualitative study used adolescents’ own experiences and perspectives of school context and wellbeing at school. Our results provide detailed information regarding specific factors of school environment and their contribution to adolescents’ wellbeing at school. This, may have several implications for practice. First, improvement of the school administration and organisation with focus on dealing with arising issues that may pose a threat to students may be beneficial for creating a safer environment and prevent similar events from happening in the future. Second, enhancement of the overall physical environment and looks of the school and classrooms and investment into more modern and utility equipment may help adolescents feel homelier and cosy at school and improve their overall impressions of the school environment.

Next, bolstering teachers’ interpersonal skills and allowing them to maintain healthy relationships with adolescents based on mutual trust and respect and providing adolescents with activities that would strengthen their bonds with peers within the class group may be great sources of resilience that would help adolescents cope and manage struggles following from the demands of school context.

### Conclusions

We identified three main groups of environmental, individual and interpersonal determinants of adolescents’ wellbeing at school according to their own school experiences. Adolescents may considerably benefit from organisational and administrative schemes adjusted to dealing with issues and boosting the relationships with teachers and peers. These may affect their consequent attitudes, academic results, engagement and professional trajectory in the future.
